# An fMRI study of finger movements in children with and without dyslexia

**DOI:** 10.3389/fnins.2023.1135437

**Published:** 2023-05-18

**Authors:** Ted K. Turesky, Megan M. Luetje, Guinevere F. Eden

**Affiliations:** Center for the Study of Learning, Georgetown University Medical Center, Washington, DC, United States

**Keywords:** connectivity, dyslexia, finger, motor, reading, reading disability

## Abstract

**Introduction:**

Developmental dyslexia is a language-based reading disability, yet some have reported motor impairments, usually attributed to cerebellar dysfunction.

**Methods:**

Using fMRI, we compared children with and without dyslexia during irregularly paced, left or right-hand finger tapping. Next, we examined seed-to-voxel intrinsic functional connectivity (iFC) using six seed regions of the motor system (left and right anterior lobe of the cerebellum, SM1 and SMA).

**Results:**

A whole-brain task-evoked analysis revealed relatively less activation in the group with dyslexia in right anterior cerebellum during right hand tapping. For iFC, we found the group with dyslexia to have greater iFC between the right SM1 seed and a medial aspect of right postcentral gyrus for left hand tapping; and greater iFC between the left SM1 seed and left thalamus, as well as weaker local iFC around the left SM1 seed region for right hand tapping. Lastly, extracted activity and connectivity values that had been identified in these between-group comparisons were not correlated with measures of reading.

**Discussion:**

We conclude that there are some aberrations in motor system function in children with dyslexia, but these are not tied to reading ability.

## 1. Introduction

Dyslexia is a learning disability characterized by slow and/or inaccurate reading of words, despite normal intelligence and/or instruction (Lyon et al., 2003). It occurs in roughly 5–12% of the population (Katusic et al., 2001). A commonly observed deficit in dyslexia is a weakness in skills collectively referred to as phonological awareness; these are needed for accurate grapheme-phoneme mapping and the successful decoding of words (Lyon et al., 2003; Peterson and Pennington, 2012). Phonological awareness plays a contributing role in bringing about skilled reading in all children (Wagner and Torgesen, 1987) and intensive instruction in this domain helps to improve reading ability in those with dyslexia (Alexander et al., 1991). These findings have jointly reinforced strong support of a phonological deficit theory for dyslexia (Stanovich, 1988). However, there are other facets of language skills and also non-language functions that have also been considered in the context of this detrimental learning disability.

Some studies have focused on motor impairments in children with dyslexia, especially for tasks that are associated with cerebellar function. For example, a study by Fawcett et al. (1996) assessed children with dyslexia on a battery of motor tasks, including tests of posture, balance, muscle tone, and complex movements, and found that they performed significantly worse on all tests compared with their age-matched controls across a range of ages (10, 14 and 18 years of age on average). Also, the investigators reported that these impairments across all tasks manifested in 74% of children with dyslexia, compared to 16% of the controls (Fawcett et al., 1996). As deficits on these types of tests are indicative of abnormalities in the cerebellum, this study and others like it, as well as a brain imaging study on motor learning (described below), have led to the theory that dyslexia may be associated with abnormalities in the cerebellum (Nicolson et al., 2001).

Yet, others have raised concerns that these “cerebellar” impairments are not reliably identified. For example, a meta-analysis quantitatively summarizing 17 studies that compared performance of balance tasks in children, adolescents, and young adults with and without dyslexia, revealed that effect sizes were not correlated with the degree of reading impairment; they were, however, correlated with whether the studies eliminated participants with attention-deficit-hyperactive disorder (ADHD; Rochelle and Talcott, 2006). Further, a study employing a battery of cerebellar tests used by Fawcett et al. (1996), this time in a group of children with ‘pure’ dyslexia (no ADHD or developmental coordination disorder) found that only 42% of these children (compared to 74% reported by Fawcett et al., 1996) performed worse than controls (Ramus et al., 2003). Ramus and colleagues concluded that motor deficits (measured, for instance, with finger-to-thumb opposition movements, bead threading, and balance tasks) are restricted to a minority of those with dyslexia, a finding that is consistent with another study (White et al., 2006). As such, the evidence that motor deficits are prevalent in children with dyslexia is mixed at best.

Turning to brain imaging studies, there is an abundance of research showing that the brain regions involved in reading and reading-related tasks are altered anatomically and functionally in dyslexia (for a review, please see Eden et al., 2016). Specifically, meta-analyses have shown left temporo-parietal, occipito-temporal, and inferior-frontal cortices to have less gray matter volume (GMV; Linkersdörfer et al., 2012; Richlan et al., 2013; Eckert et al., 2016) and less activation during reading-related tasks (Maisog et al., 2008; Richlan et al., 2011; Linkersdörfer et al., 2012) in dyslexia. By comparison, only some meta-analyses have shown differences in brain regions associated with motor function and/or the cerebellum in dyslexia. Reduced GMV has been reported in dyslexia in bilateral posterior cerebellum lobule VI (Linkersdörfer et al., 2012; Stoodley, 2014) and right posterior cerebellum (Eckert et al., 2016), while another meta-analysis reported no differences in cerebellar GMV (Richlan et al., 2013). However, posterior cerebellum, and specifically lobule VI, is associated with language processing and is distinct from the anterior, motor aspect of cerebellum, offering little evidence in support of a specific motor system dysfunction. Of interest is an empirical study that reported anomalies of the cerebellum (anterior and posterior extents) for brain neurochemistry (Rae et al., 1998). However, no meta-analyses on reading or reading-related tasks reported less activation in the cerebellum in dyslexia relative to controls (Maisog et al., 2008; Richlan et al., 2011) with one reporting *more* in the left cerebellum in dyslexia (Linkersdörfer et al., 2012). One meta-analysis report identified more activity in left precentral gyrus in dyslexia and attributed these to compensatory articulatory processes (Richlan et al., 2011). While there are numerous studies on brain activity during reading and reading-related tasks in dyslexia (as used for these meta-analyses), there are only two neuroimaging studies that have examined motor tasks in dyslexia, specifically. Nicolson et al. (1999) examined right hand finger movement sequences, both pre-learned and novel, in adults with and without dyslexia. They found that for both pre-learned and new sequences, activation measured with positron emission photography (PET) was less in the right cerebellum in the group with dyslexia compared to controls. Further, for the new sequence task, adults with dyslexia showed greater activation in right medial prefrontal cortex and parts of bilateral temporal and parietal cortices (Nicolson et al., 1999). In a functional magnetic resonance imaging (fMRI) study, Menghini et al. (2006) found that for pseudorandom and repeating finger movement sequences, adults with dyslexia showed *more* activation in right cerebellum lobule VI, right lateral premotor cortex, and bilateral inferior parietal cortex compared to controls (Menghini et al., 2006). As such, the corpus of studies is small and inconsistent (one showing relatively less, and the other relatively more activity in the cerebellum in dyslexia). While the notion of a motor deficit in dyslexia is considered to be controversial, further research is warranted given implications for treatment of dyslexia targeting the motor system (Reynolds et al., 2003).

In the current study we examined activity during thumb movements of the left and right hands (in separate runs) and compared these between groups of children with and without dyslexia. Studies in children are important as those in adults, such as those described above, leave open the possibility that any difference observed may be a consequence of having had dyslexia for several years rather than reflecting the cause of the disability. Further, the group with dyslexia was matched to the control group on a measure of ADHD symptoms, since ADHD may contribute to motor deficits in dyslexia (Rochelle and Talcott, 2006). Based on the two brain imaging studies on motor movement in adults with dyslexia described above (Nicolson et al., 1999; Menghini et al., 2006), one might expect children with dyslexia to exhibit greater activity in regions subserving voluntary movement (e.g., premotor cortices). One might also expect activation differences in the cerebellum, although prior findings are conflicting about hyper- or hypo activity in the cerebellum in dyslexia. Given the extensive connections within the motor system, especially those between the cerebellum and cerebrum (Balsters et al., 2014), we also examined intrinsic functional connectivity (iFC) to capture correlations in these regions’ brain activity using background connectivity (Al-Aidroos et al., 2012; Norman-Haignere et al., 2012; Wang and Voss, 2014; Gratton et al., 2016). IFC was measured between seed regions (located in the cerebellum and motor cortex) with the rest of the brain (seed-to-voxel iFC) as a way to allow for the interpretation of the activation results in the context of functional connections, including functional connections not specific to the task. Finally, the signal from any resulting activations or functional connections identified to be different between the two groups were then tested for correlations with reading ability. The ultimate goal was to determine whether there are differences in the integrity of the motor system in dyslexia and for any aberrations found, to test for a direct relationship with reading.

## 2. Methods

### 2.1. Participants

Children with dyslexia and their age-matched controls were part of a larger program of research on reading disability. All children were right-handed (i.e., with scores above 30 on the Edinburgh Handedness Inventory; Oldfield, 1971), monolingual English speakers without prior diagnoses of neurological disorders. The children with and without dyslexia were recruited from comparable geographic regions within the greater Washington, DC metropolitan area and from families at comparable socioeconomic standings. The children with dyslexia came from a private school specializing in learning disabilities. Nine of the children in the control group were included in a prior functional study comparing the motor system between children and adults (Turesky et al., 2018) and others were included in prior functional studies not involving the motor system (Olulade et al., 2013, 2015; Evans et al., 2014a,b; Ashburn et al., 2020).

The children with dyslexia had a documented history of underachievement in reading. To evaluate reading skills, all children underwent the Woodcock**-**Johnson Tests of Achievement (Woodcock et al., 2001). The children with dyslexia were included if they performed <92 on the Word Identification subtest of the Woodcock**-**Johnson Tests of Achievement, while those in the control group had a score of > 92 (30th percentile). The Word Identification subtest requires the naming of letters and words of increasing difficulty aloud from a list and is untimed. The reading fluency test requires participants to silently read sentences and indicate whether they are true or false within 3-min. We assessed intelligence quotient (IQ) in all participants using the Wechsler Abbreviated Scale of Intelligence (Wechsler, 1999) to ensure all children had Full-Scale IQs at or above 80.

The iFC analyses (as described below) required strict criteria for rejecting scans with head motion, leading to the elimination of 15 children with dyslexia and 16 typically reading children. To match our two groups on Conners’ ADHD index T-scores and chronological age, we removed another five participants. The final groups comprised 15 children in the group with dyslexia (mean age: 10.00 ± 1.3 years) and 15 children in the control group (mean age: 8.72 ± 2.0 years) with a large discrepancy in their reading skills. Demographic information for the final sample is summarized in Table 1.

**Table 1:** Participants’ demographics.

	Control	Dyslexia	value of *p*
*N*	15	15	
Sex (F/M)	7/8	7/8	
Age (years)	8.72 (2.0)	10.0 (1.3)	ns
Range (years)	7.1–13	7.4–12	
Edinburgh handedness inventory	78.6 (21)	91.3 (19)	ns
Full-scale IQ	123 (14)	105 (12)	*p* < 0.001
Word identification	118 (9.8)	78.1 (10)	*p* < 0.05
Reading fluency	122 (14)	72 (14)	*p* < 0.05
Conners’ ADHD index	52 (8.6)	55 (7.7)	ns

### 2.2. MRI task and data acquisition

Participants performed visually and irregularly paced, unimanual finger tapping tasks during functional data acquisition, as used in a prior study of children and adults (Turesky et al., 2018). They were instructed to press the button with their thumb in response to a circle surrounding a cross-hair, which was omnipresent throughout the run. The tasks were presented using a block design, which consisted of 4 tapping blocks interspersed with fixations. All tapping blocks were 24 s. Within these blocks, the timing of the stimulus presentations varied and a 100 ms tapping stimulus appeared at one of three intervals: once per 650 ms, once per 900 ms, or once per 1,150 ms. Each interval was used 8 times per block and interval order was randomized and differed for each tapping block. Left and right finger tapping were performed in separate runs and each run consisted of 69 volumes (32 tapping volumes and 37 fixation volumes). For both, participants could view stimuli from inside the scanner using an angled mirror apparatus fastened to the head coil, which relayed projections from a screen behind the scanner. Participants were familiarized with the tasks and scanner environment by practicing each task in a mock scanner before entering the real scanner.

Structural MPRAGE and functional EPI images were acquired on a 3 T Siemens Trio scanner. MPRAGE scans were acquired with the following parameters: TR = 1,600 ms, TE = 4.38 ms, 160 axial slices, 1 mm^3^ voxels, FOV = 256 mm. EPI scans were acquired with blood-oxygen-level dependent (BOLD) contrasts, using TR = 3,000 ms, TE = 30 ms, 50 axial slices acquired interleaved and anterior to posterior with a 0.2 mm gap, in-plane resolution of 64 × 64 (3 mm isotropic voxels), and 192 mm FOV.

### 2.3. In-scanner behavior data analysis

We calculated two performance measures collected during the scan: (1) accuracy, defined as the percent of trials in which participants pressed the button when prompted; and (2) response times, defined as the time between the onset of the circle around the fixation cross and the button press by the participant. Data files were lost for two participants with dyslexia and so group-level performance statistics were calculated without them.

### 2.4. MRI data analyses

#### 2.4.1. Preprocessing and head motion quality control

All preprocessing steps were performed using MATLAB 2016a (*MathWorks*) and SPM12.^1^

MPRAGE: For all participants, images were warped into Montreal Neurological Institute (MNI) stereotaxic space to correct for inter-subject variability and segmented into gray matter, white matter and cerebrospinal fluid (CSF) masks using the VBM8 toolbox.

EPI: To reduce T1 saturation effects, we discarded the first 3 volumes from each run; we also discarded the final two volumes, leaving 64 volumes. Preprocessing comprised five major steps: (1) slice time correction to account for sampling superior and inferior parts of the brain at different times, (2) realignment of scans to correct for inter-scan head motion throughout the run, (3) coregistration to the native space MPRAGE images, (4) deformation to warp EPI images into MNI space using the subject-specific transformations applied to the MPRAGE images, and (5) smoothing with 8.0 mm FWHM Gaussian kernel to improve the signal-to-noise ratio. Following preprocessing, smoothed images were overlain with the MNI template to ensure successful normalization.

We undertook several additional steps to account for in-scanner head motion, since functional connectivity in general (Van Dijk et al., 2012; Satterthwaite et al., 2013) and especially in children (Satterthwaite et al., 2012) suffers considerably from head motion artifacts. First, we removed participants for whom ≥ 20% of the total number of scans from either their left or right hand runs were preceded by inter-scan head motion greater than 0.75 mm (25% of the voxel size) root-mean-square (RMS) displacement (i.e., d^2^ = ∆x^2^ + ∆y^2^ + ∆z^2^ + [(65π/180)^2^ · (∆pitch^2^ + ∆rol^2^ + ∆yaw^2^)]; Mazaika et al., 2005). This procedure removed 16 children from the control group and 15 from the group with dyslexia. The remaining participants (15 children in each group) did not differ on the percentage of scans removed from each participant’s dataset for the left [*t*(28) = −0.18; *p* > 0.05] or right hand [*t*(28) = −0.44; *p* > 0.05; Table 2]. Second, scans that were preceded by ≥ 0.75 mm inter-scan head motion were entered as regressors at the first level (please see below). Third, we performed two-sample t-tests on mean and maximum inter-scan RMS displacement (after removing scans preceded by ≥ 0.75 mm head motion; Power et al., 2012; Alaerts et al., 2014) and found no significant differences for left (mean interscan: *t*(28) = −0.24; *p* > 0.05; max interscan: *t*(28) = −0.31; *p* > 0.05) or right hand tapping data [mean interscan: *t*(28) = −0.011; *p* > 0.05; max interscan: *t*(28) = 1.65; *p* > 0.05], as seen in Table 2. And fourth, we assessed stimulus-correlated motion using Artifact Detection Tools (ART; adjusted in-house)^2^ to display correlation coefficient *r*-values for each translation and rotation parameter, because stimulus-correlated motion has been shown to reduce sensitivity of first-level effects (Johnstone et al., 2006). A MANOVA on these Fisher *r*-to-*z* transformed values showed no significant difference between groups on these measures.

**Table 2:** Motion description.

	Control	Dyslexia	value of *p*
L. % movement scan	3.23 (5.7)	3.64 (6.7)	ns
L. mean interscan displacement (mm)	0.138 (0.058)	0.142 (0.047)	ns
L. max interscan displacement (mm)	0.469 (0.16)	0.484 (0.11)	ns
L. stim correlated motion (dim. pool)	0.0729 (0.051)	0.0782 (0.068)	ns
R. % movement scan	3.44 (4.9)	2.61 (5.2)	ns
R. mean interscan displacement (mm)	0.151 (0.047)	0.151 (0.046)	ns
R. max interscan displacement (mm)	0.604 (0.11)	0.516 (0.17)	ns
R. stim correlated motion (dim. pool)	0.0520 (0.048)	0.0964 (0.073)	ns

#### 2.4.2. Whole-brain activation analyses

Activation analyses for the whole brain were performed in SPM12. For each participant, first-level statistics were performed by first applying a temporal high pass filter of 128 s, and then modeling each condition (left- and right-hand tapping) with a convolution of the canonical hemodynamic response function and our experimental block design. Fixation was treated as baseline, rather than as a distinct condition. We used an autoregressive (AR 1) model to reduce serial correlations from biorhythms and unmodeled neuronal activity. To account for head motion as a confound of the button press and for changes in the global mean signal, we created a multiple regression model comprising the RMS displacement from origin, a logical vector to indicate whether a particular scan was preceded by ≥ 0.75 mm motion, and the global mean signal at each time point. This procedure generated within-subject beta maps for left and right hand tapping separately.

To identify within-group activations for the whole brain, we performed one-sample t-tests for left as well as right hand tapping, and both for the control group and the group with dyslexia. To identify between-group activation differences, we performed two-sample t-tests for control > dyslexia and dyslexia > control contrasts using left as well as right hand tapping data. We applied SPM’s EPI.nii template as an explicit mask. Although age was not significantly different between groups, it was trending at *p* = 0.056. Therefore, we entered age as a covariate of no interest in the between-group comparison. All clusters output from these second-level analyses were reported as significant using an FDR cluster-level correction of *p* < 0.05 and a height threshold of *p* < 0.001. Percent signal changes (PSCs) were extracted from participants’ first level tapping data for clusters significant in the between-group comparison using MarsBaR 0.44.^3^

#### 2.4.3. Seed-to-voxel intrinsic functional connectivity analyses

For the functional connectivity analyses, we constructed seed regions from the activation maps, consistent with earlier work (Rehme et al., 2013). Seed regions were derived by overlaying the activation results from the groups with and without dyslexia. To this end, we first identified those voxels that were active during each task (left- and right-hand tapping) in both groups (control group and the group with dyslexia) and then generated group overlap regions for these. Three regions resulted in each hemisphere, namely the cerebellum, primary sensory motor cortex (SM1), and supplementary motor area (SMA), resulting in three seed regions in each hemisphere. Then, 6 mm radius spheres were built around the centers of mass for these regions, located as follows: left cerebellum anterior lobe (*x* = −18, *y* = −56, *z* = −17), right cerebellum anterior lobe (*x* = 13, *y* = −55, *z* = −15), left SM1 (*x* = −37, *y* = −22, *z* = 60), right SM1 (*x* = 38, *y* = −22, *z* = 61), left SMA (*x* = −2.6, *y* = −2.5, *z* = 61), and right SMA (*x* = 2.3, *y* = −1.8, *z* = 60). Defining seeds based on overlapping activation of the two groups prevented bias toward either group and defining seeds by independent sources would have introduced bias in favor of the control group.

All iFC analyses were performed using CONN 15 h/16a (Whitfield-Gabrieli and Nieto-castanon, 2012).^4^ To maximize signal-to-noise, all unsmoothed functional data (following preprocessing and motion quality control steps detailed earlier) were denoised, which involved simultaneous regression of temporal confounding factors and temporal filtering. Temporal confounding factors included the six rigid body head position parameters, a logical vector to indicate scans that were preceded by inter-scan head motion ≥ 0.75, and block conditions (fixation, left hand tapping, right hand tapping) convolved with the canonical hemodynamic response function. CONN also implements the CompCor method (Behzadi et al., 2007; Chai et al., 2012), in which five principal components were estimated from the subject-specific white matter and CSF masks generated in the VBM segmentation step above. We did not use temporal derivatives, as they decreased the signal-to-noise ratio. We used a band-pass filter of 0.008–0.090 Hz for our iFC analysis for which block-to-block discrimination is not necessarily desired (please see footnote 4 forum for a discussion of block-to-block discrimination); our low-pass threshold is consistent with that of previous background iFC studies (Rehme et al., 2013; You et al., 2013) and a range similar to this one has been advocated for reducing iFC measures related to motion (Satterthwaite et al., 2013). These steps produced denoised, residual BOLD time series for every gray matter voxel for every participant.

Seed time series were computed by averaging denoised BOLD time series across the voxels within the seed. Maps generated for the right cerebellum, left SM1, and left SMA seeds were based on right hand tapping data; and maps generated for the left cerebellum, right SM1, and right SMA seeds were based on left hand tapping data.

Bivariate correlations were computed from time series data from the full runs for each hand. Here, first-level analyses were performed using weighted GLM and HRF weighting. This resulted in single-subject *r*-maps in which the value of each voxel represents the correlation coefficient of that voxel’s denoised time series with the seed time series. These maps were subsequently transformed into *z*-maps using Fisher’s *r*-to-*z* transform.

Generation of within-group brain maps used one-sample t-tests for each group and seed separately. A positive iFC value at a given voxel indicated that that voxel’s denoised time series was correlated with the time series of the seed. A negative iFC value at a given voxel indicated that that voxel’s time series was anti-correlated with the time series of the seed. Generation of between-group brain maps used two-sample t-tests for controls > dyslexia and dyslexia > controls contrasts for each seed separately. Again, age was entered as a covariate of no interest in between-group comparisons. All clusters output from these second-level analyses were reported significant using an FDR cluster-level correction of *p* < 0.05 and a height threshold of *p* < 0.001.

To differentiate positive and negative within-group iFC and to examine inter-subject variability in the between-group results, we extracted single-subject iFC measures from all resulting between-group clusters for both contrasts (control > dyslexia and dyslexia > control) using the REX toolkit.^5^

#### 2.4.4. Brain-behavior relationships

Finally, to determine if there were relationships for (i) activation with reading ability and (ii) functional connectivity with reading ability in those regions where the groups differed, we performed Partial (Pearson) correlations between individual PSC and iFC estimates extracted from clusters that were significant in the between-group comparisons and measures of reading: the Word Identification and the Reading Fluency subtests of the Woodcock**-**Johnson Tests of Achievement (Woodcock et al., 2001). Because the data were binomially distributed for reading scores (according to group), we controlled for the effect of group using partial correlations, as done in previous studies (Schurz et al., 2014).

#### 2.4.5. Reporting and visualization of MRI results

Significant clusters’ peak voxel MNI stereotaxic coordinates and voxel extents were reported using SPM12 and CONN 15 h/16a, converted into Talairach anatomical space (Talairach and Tournoux, 1988) using the *icbm2tal* algorithm (Lancaster et al., 2007) included in GingerALE 2.3.6, and labelled as anatomical regions according to the Talairach Client 2.4.3.^6^ Any peak coordinate greater than 11 mm from gray matter according to the Talairach Client was investigated using the Talairach Applet,^7^ and a visual determination of its anatomical location was made.

Cortical motor regions (e.g., SM1, SMA, etc.) were labelled by overlaying thresholded brain maps on the Human Motor Area Template [HMAT; (Mayka et al., 2006)].^8^ This template was derived by implementing the ALE method on 126 fMRI or PET studies involving motor control, and it demarcates the motor areas into 3 main divisions (SM1, medial premotor cortex – MPMC, and lateral premotor cortex – LPMC) and subdivisions (primary motor and somatosensory cortices for SM1, SMA and pre-SMA for MPMC, and ventral premotor cortex – PMv – and dorsal premotor cortex – PMd – for LPMC). For our reporting, we differentiated the subdivisions of MPMC and LPMC, but not SM1, because most of the effects in this region registered as a single cluster. This is different from activations in MPMC and LPMC, which often registered in one but not both subdivisions. All results were visualized using the Mango software package^9^ with the Colin brain template in MNI space (Holmes et al., 1998). All voxels at surface depth ≤ 10 voxels are visualized at the surface.

## 3. Results

### 3.1. Behavioral results

Group averages for in-scanner head motion and performance (accuracy and response time) are summarized in Tables 2, 3, respectively. No significant differences were observed for head motion nor for performance between the control group and the group with dyslexia.

**Table 3:** In-scanner performance.

	Control	Dyslexia	value of *p*
L. accuracy (% correct)	0.88 (0.1)	0.85 (0.08)	ns
L. response time (ms)	324 (40)	328 (35)	ns
R. accuracy (% correct)	0.88 (0.1)	0.84 (0.1)	ns
R. response time (ms)	334 (55)	341 (44)	ns

### 3.2. Whole-brain activation analyses

#### 3.2.1. Within-group maps

**Figure 1:**
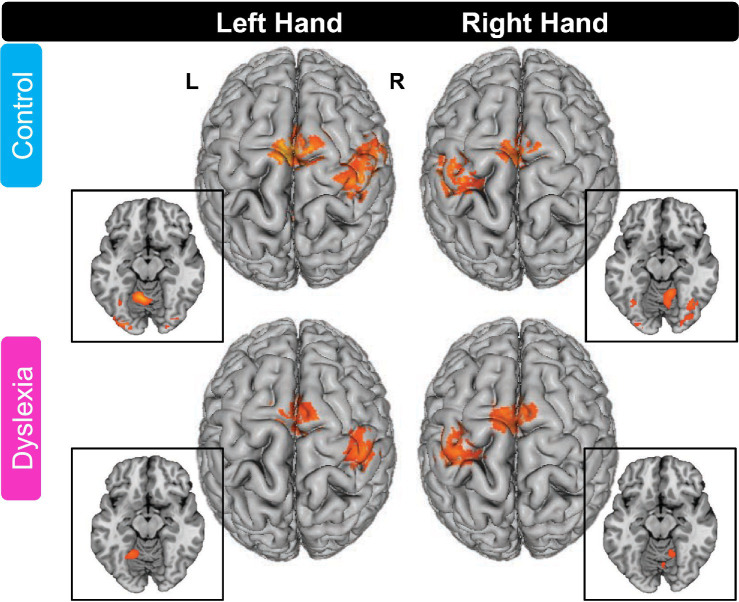
Whole-brain activation maps for finger tapping in the control group and the group with dyslexia. For both groups, left and right finger tapping relative to fixation baseline elicited activations in cortical and subcortical (not shown) brain areas (FDR cluster-level corrected threshold of *p* < 0.05). All axial slices in insets are at z = −15 (MNI). L, left hemisphere; R, right hemisphere. Please see Table 4 for peak locations.

Whole-brain activation maps for the groups with and without dyslexia during tapping compared to the fixation baseline are depicted in Figure 1 and activation peaks are described in Table 4.

**Table 4:** Activation peaks for within-group contrasts for left as well as right hand tapping.

Anatomical region	Functional motor region	BA	Peak MNI coordinate			*k*	*Z*
			x	y	z		
**Control**							
*Left hand tapping*							
R. postcentral gyrus	R. SM1	3	36	-20	51	2,173	5.50
L. medial frontal gyrus	L. SMA	6	-4	-6	69	2,268	5.81
L. ant. cerebellum			−9	−56	−10	3,748	6.57
R. claustrum			34	16	9	386	4.43
R. midbrain			9	−24	−9	425	4.05
*R. inferior* occipital gyrus		18	27	−93	−4	1,639	5.07
*Right hand tapping*							
L. precentral gyrus	L. SM1	4	−30	−24	57	1,629	5.57
L. medial frontal gyrus	L. SMA	6	−4	−8	62	1,262	5.67
R. ant. cerebellum			14	−54	−20	4,908	5.22
L. post. cerebellum			−40	−64	−24	503	4.22
L. thalamus			−10	−22	2	760	4.70
L. inferior occipital gyrus		18	−32	−94	−4	1,211	5.23
**Dyslexia**							
*Left hand tapping*							
R. postcentral gyrus	R. SM1	3	40	−24	58	1,079	4.77
L. medial frontal gyrus	L. SMA	6	−4	0	58	1787	4.64
*R. insula*	R. PMv	13	50	−2	9	184	3.68
L. ant. cerebellum			−21	−56	−16	646	4.46
R. thalamus			16	−20	8	198	4.25
R. lingual gyrus		17	24	−96	3	419	4.64
*Right hand tapping*							
L. postcentral gyrus	L. SM1	3	−38	−26	63	1,154	4.76
L. medial frontal gyrus	L. SMA	6	−9	−2	64	1,268	4.73
R. ant. cerebellum			15	−56	−15	503	3.82
L. thalamus			−9	−21	9	539	4.46
R. middle occipital gyrus		18	39	−92	0	249	4.40

##### 3.2.1.1. Control group

In the control group, left hand thumb tapping induced activation in right SM1 (extending into right PMd), left SMA (extending into right SMA and anteriorly into bilateral pre-SMA), left anterior cerebellum, right claustrum (extending into right PMv), right midbrain (extending into right thalamus), and right inferior occipital gyrus.

Right-hand thumb tapping was associated with activation in left primary sensorimotor cortex (SM1), left supplementary motor area (SMA; extending into right SMA and bilateral pre-SMA), right anterior cerebellum, left posterior cerebellum, left thalamus (extending into right thalamus), and left inferior occipital gyrus.

##### 3.2.1.2. Group with dyslexia

In the group with dyslexia, left hand thumb tapping was associated with activation in right SM1, left SMA (extending into right SMA and bilateral pre-SMA), right PMv (though the peak was located in right insula, not precentral gyrus), left anterior cerebellum, right thalamus, and right lingual gyrus.

Right hand thumb tapping showed activation in left SM1, left SMA (extending into bilateral pre-SMA), right anterior cerebellum, left thalamus, and right middle occipital gyrus.

#### 3.2.2. Between-group maps

**Figure 2:**
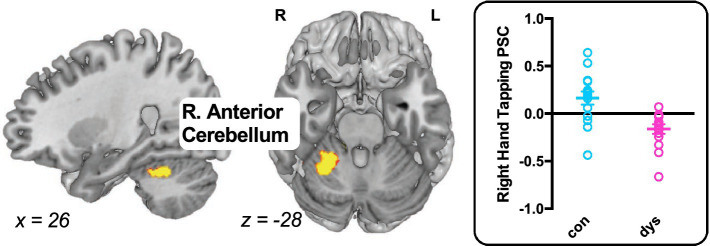
Differences in task-evoked activation between the control group and the group with dyslexia. The group with dyslexia exhibited weaker activation during right hand tapping in right anterior cerebellum compared with controls. Brain maps *p* < 0.05 FDR cluster-level corrected. Individual percent signal change (PSC) extracted from the right anterior cerebellum are depicted to the right of the brain map for showing inter-subject variance. L, left hemisphere; R, right hemisphere.

For left hand tapping, no significant differences were observed between the control group and the group with dyslexia. For right hand tapping, relatively less activation was observed in right anterior cerebellum in the group with dyslexia (*x* = 26, *y* = −52, *z* = −28; *k* = 463; *Z* = 3.97; Figure 2), and there were no areas that had relatively more activity in the group with dyslexia.

### 3.3. Seed-to-voxel intrinsic functional connectivity analyses

#### 3.3.1. Within-group maps

We performed bivariate correlation analyses to identify brain areas exhibiting functional connectivity with any of the six seed regions (left and right cerebellum anterior lobe, SM1, and SMA). For brevity we do not report on significant self-connections, which are connections that are close to the seed (e.g., if, when using a left cerebellum seed, a cluster emerges with a peak coordinate in left cerebellum). Additionally, unless otherwise specified, iFC estimates are positive.

##### 3.3.1.1. Control group

For the left-hand tapping run, the control group exhibited positive iFC between the left cerebellum seed and right SM1; and between the left cerebellum seed and right SMA (extending into left SMA). There was also iFC between the right SM1 seed and left anterior cerebellum. Lastly, there was iFC between the right SMA seed and left SM1 (extending into PMd); between the right SMA seed and left PMv (two separate clusters); and between the right SMA seed and left lentiform nucleus. The control group also exhibited negative iFC between the right SMA seed and right MTG; between the right SMA seed and left MTG; and between the right SMA seed and left IFG.

**Figure 3:**
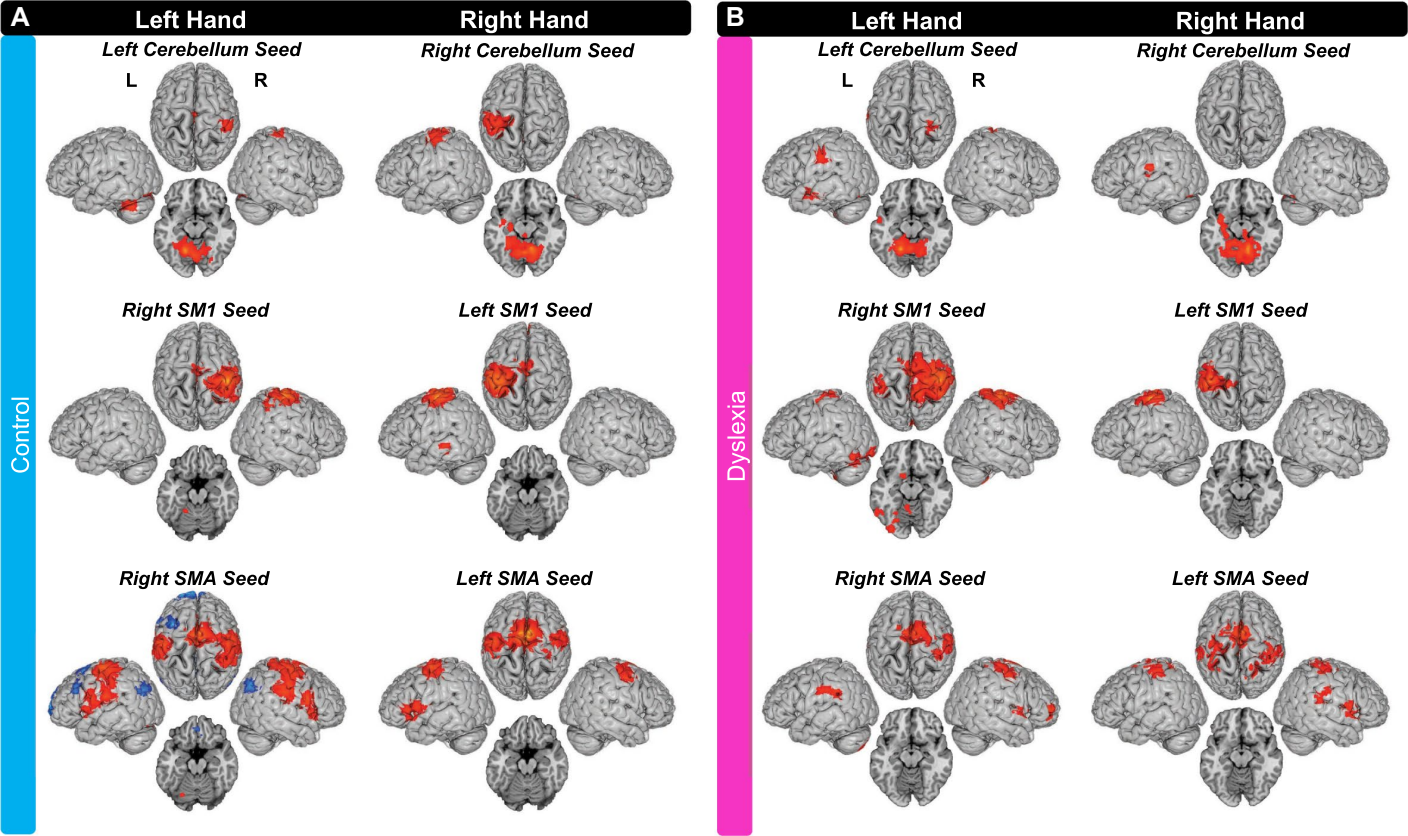
Seed-to-Voxel Intrinsic Functional Connectivity for **(A)** the control group and **(B)** the group with dyslexia. IFC with seeds in left cerebellum, right SM1, and right SMA during left finger tapping data; and iFC with seeds in right cerebellum, left SM1, and left SMA during right finger tapping data. Axial slices are *z* = −15 (MNI) for cerebellum seeds and *z* = −20 (MNI) for SM1 and SMA seeds. All maps were thresholded with FDR cluster-level correction of *p* < 0.05. L, left hemisphere; R, right hemisphere. Supplementary Table S1 provides the full list of brain areas with iFC to these seeds.

For right-hand tapping, the controls exhibited positive iFC between the right cerebellum seed and left SM1 (though the anatomical location was left inferior parietal lobule and not postcentral gyrus; extending into PMd). There was also iFC between the left SM1 seed and left SMA (extending into right SMA and bilateral pre-SMA); between the left SM1 seed and left MTG; between the left SMA seed and right SM1; and between the left SMA seed and right PMd (extending into PMv; Figure 3A).

##### 3.3.1.2. Group with dyslexia

For the left-hand tapping run, the group with dyslexia exhibited positive iFC between the left cerebellum seed and left SM1; between the left cerebellum seed and right SM1; and between the left cerebellum seed and left MTG. There was also iFC between the right SM1 seed and left SM1 (two separate clusters), between the right SM1 seed and left anterior cerebellum, and between the right SM1 seed and left fusiform gyrus. Lastly, there was iFC between the right SMA seed and right SM1 (two separate clusters with the more lateral extending into PMd); and between the right SMA seed and left STG.

For the right-hand tapping run, the group with dyslexia exhibited positive iFC between the right cerebellum seed and left superior temporal gyrus (STG), but not between this seed and any motor regions (as defined by HMAT; please see methods for details). There was no iFC between the left SM1 seed and other regions. Lastly, the group with dyslexia exhibited iFC between the left SMA seed and right SM1, between the left SMA seed and left SM1 (though the anatomical peak was outside pre/postcentral gyrus), between the left SMA seed and left PMv, between the left SMA seed and right STG, and between the left SMA seed and right inferior parietal lobule (Figure 3B).

#### 3.3.2. Between-group differences

**Figure 4:**
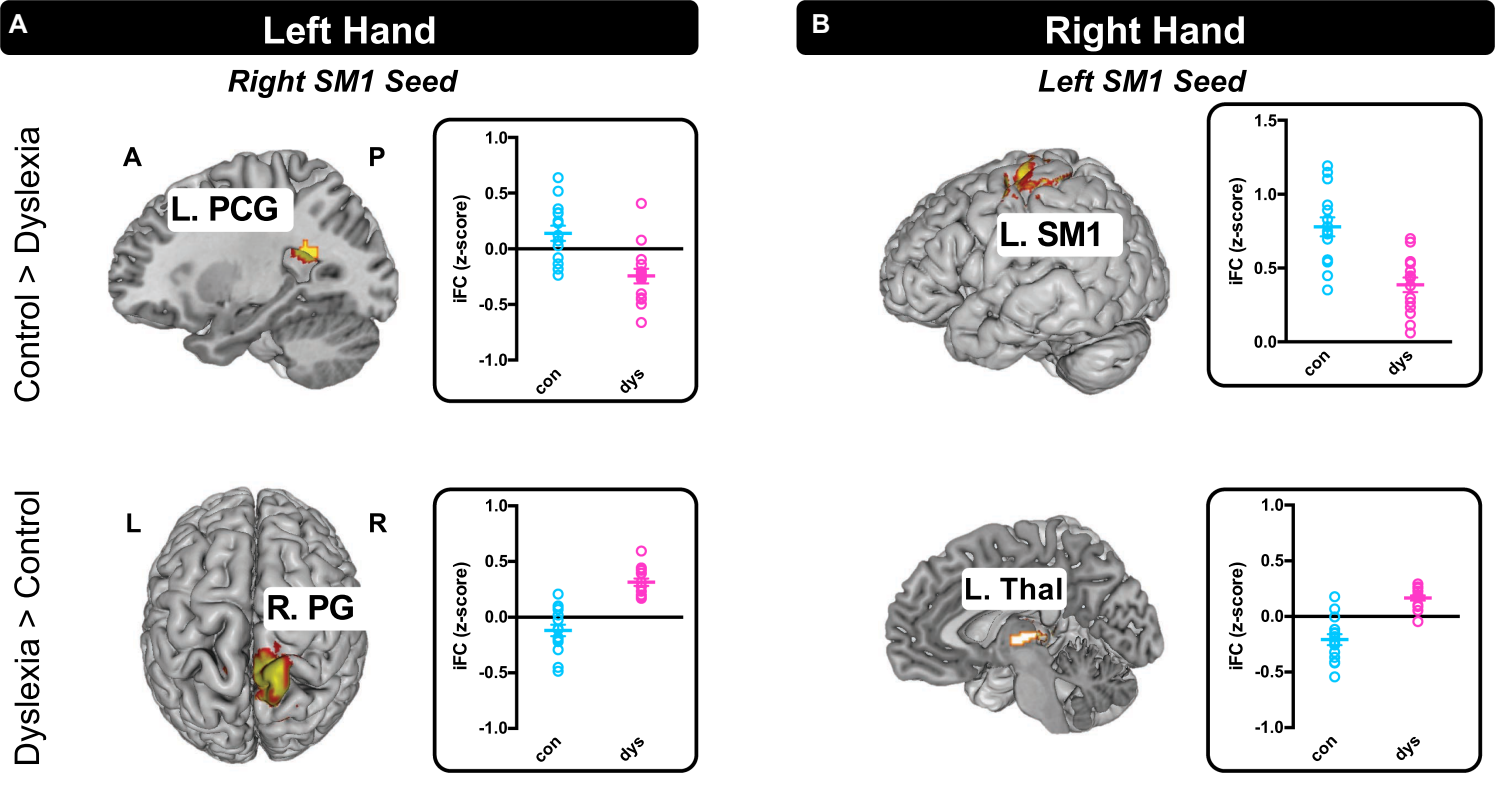
Seed-to-Voxel Intrinsic Functional Connectivity differences between the control group and the group with dyslexia. **(A)** There were two iFCs that differed between the groups during left hand tapping and **(B)** two during right hand tapping. All maps *p* < 0.05 FDR cluster-level corrected. Individual iFC measures extracted from significant clusters are depicted to the right of the brain map for differentiating positive and negative within-group iFC and showing inter-subject variance. L, left hemisphere; R, right hemisphere; A, anterior; P, posterior; SM1, primary sensorimotor cortex; PCG, posterior cingulate gyrus; PG, postcentral gyrus; Thal, thalamus.

Figure 4 shows the results by seed regions during (A) left-hand and (B) right-hand tapping, as well as the individual iFC measures extracted from significant clusters shown for each group. For left hand tapping, the group with dyslexia exhibited weaker iFC than controls between the right SM1 seed and an area nearby left posterior cingulate gyrus (*x* = −26, *y* = −52, *z* = 20; *k* = 97; *Z* = 3.91). The group with dyslexia also exhibited greater iFC between the right SM1 seed and the medial aspect of right postcentral gyrus (*x* = 8, *y* = −38, *z* = 82; *k* = 119; *Z* = 4.52) (Figure 4A).

For right hand tapping, the group with dyslexia compared with the controls exhibited relatively weaker local iFC around the left SM1 seed (*x* = −36, *y* = −26, *z* = 50; *k* = 121; *Z* = 4.44). Also, the group with dyslexia exhibited greater iFC between the left SM1 seed and left thalamus (*x* = −4, *y* = −18, *z* = 2; *k* = 83; *Z* = 4.79) (Figure 4B).

#### 3.3.3. Brain-behavior relationships

We tested relationships for (i) functional activation with reading ability and (ii) functional connectivity with reading ability. Specifically, we extracted individual PSC from the right anterior cerebellar region identified to be different in activation between the two groups and correlated it with both measures of reading (Word Identification and Reading Fluency). Next, we extracted iFC estimates from the functional connections that differed between the two groups (right SM1 seed with posterior cingulate, right SM1 seed with medial aspect of right postcentral gyrus, left SM1 with left thalamus, and left SM1 with surrounding regions) and correlated these with both measures of reading, too. Because the data were binomially distributed for reading scores (according to group), we controlled for the effect of group using partial correlations, and we applied a Bonferroni correction for the multiple tests. No brain-behavior partial correlations were significant.

## 4. Discussion

This is the first study to jointly investigate activity and functional connectivity of the motor system in dyslexia. Visually paced, unimanual finger tapping with either hand engaged brain regions as expected based on prior studies on motor movement for each group (Witt et al., 2008): SM1 contralateral to the side of movement, bilateral SMA, and anterior cerebellum ipsilateral to the side of movement. Between-group comparisons showed that the group with dyslexia exhibited less activation than the control group in right anterior cerebellum. When examining (seed-to-voxel) intrinsic functional connectivity (iFC) using three seed regions in both hemispheres (cerebellum, SM1, and SMA), both groups overall exhibited functional connections between regions of the cerebral cortex, as well as between the cerebral cortex and the cerebellum. Between-group comparisons revealed four differences in iFC, all of which involved SM1 and connections with left hemisphere brain regions. However, none of these regions differing between the two groups in activity or functional connectivity correlated with measures of reading ability (accuracy or fluency). Overall, these findings suggest some differences in activity and intrinsic functional connectivity in dyslexia that are not directly tied to reading.

### 4.1. Functional anatomy of motor movements during finger tapping

Several studies have examined motor system activation in typically developing children (Rivkin et al., 2003; Vandermeeren et al., 2003; Mostofsky et al., 2006; De Guio et al., 2012; Roessner et al., 2012, 2013; Turesky et al., 2018) with many more studies having been conducted in adults (please see Witt et al., 2008, for a meta-analysis). As in the present study, previous studies in children have reported activation of SM1, SMA, and cerebellum. Overall, the groups with and without dyslexia showed patterns of activation during finger tapping that are consistent with previous studies in typically developing children and with the functional anatomy underlying finger tapping in general.

Turning to functional connectivity, de Bie et al. (2012) characterized the motor system in children (age range: 5–8 years) as part of a resting-state (i.e., awake, no task) study on intrinsic functional connectivity of the entire brain (de Bie et al., 2012). Using an independent component analysis (ICA), the study revealed several resting-state networks, including two sensorimotor networks, one of which encompassed bilateral SM1, bilateral SMA, and bilateral cerebellum, the same regions amongst which we found functional connectivity. As such, our within-group findings are overall highly consistent with the literature.

### 4.2. Activation during motor movement in dyslexia

Two studies have compared brain activity in groups with and without dyslexia during a finger movement task, both conducted in adults (Nicolson et al., 1999; Menghini et al., 2006). The first employed a right hand, multi-digit motor sequence task with two conditions, pre-learned and novel motor sequences. For the pre-learned sequence, the group with dyslexia exhibited relatively weaker activation in right cerebellum and left cingulate gyrus; no areas were more active in the group with dyslexia. For the novel sequence task, the group with dyslexia again exhibited relatively weaker activation in the same right cerebellum region, demonstrating a difference in the cerebellum that was identified in two motor tasks, independent of whether they were pre-learned or novel. Greater activation was also observed in the group with dyslexia compared to the control group during the novel sequence in bilateral angular gyrus and left STG, as well as an area labelled as medial area 9 in BA 9 (Nicolson et al., 1999). The authors focused mostly on the cerebellum findings, arguing that these provide brain-based evidence for their early behavioral findings that were indicative of cerebellar abnormalities (Fawcett et al., 1996). The investigators used two paradigms that differed in a motor learning component, with the expectation that there would be greater activation in the cerebellum performing the pre-learned sequence relative to the new sequence in each group. However, this expectation was not met for the control group, and there were differences in activation between the two groups in the cerebellum for both sequences, thereby indicating a more general motor deficit rather than the predicted deficit in motor learning.

In the study by Menghini et al. (2006), adults performed two types of motor sequence tapping: a random sequence and a repetition of a specific sequence with the expectation that participants would implicitly learn this repeated sequence over the course of the experiment. When combining random and repeated sequences, both groups exhibited activation in bilateral cerebellum lobule VI, bilateral premotor cortex, left superior parietal lobule, and bilateral inferior parietal cortex. The control group also showed activation in left basal ganglia, left SMA, and right superior parietal lobule, while the group with dyslexia did not show activation in any additional brain areas. When comparing the groups with each other, the group with dyslexia exhibited greater activation in right cerebellum lobule VI, right lateral premotor area, and bilateral inferior parietal cortex compared with typical readers; there were no regions where the group with dyslexia showed less activation (Menghini et al., 2006). As such, the results of a right cerebellum difference in dyslexia between the two studies had opposite outcomes. Taken together there are few studies of the motor system in dyslexia and no consistent evidence in support of aberrant function or anatomy.

Our findings of weaker activity in children with dyslexia in right anterior cerebellum during right hand tapping are in line with the study by Nicolson et al. (1999), which showed weaker activity in adults with dyslexia in the right cerebellum. These findings might explain reports of worse performance in dyslexia compared with typical readers on a variety of cerebellum-specific (Fawcett et al., 1996), as well as simpler (Wolff et al., 1984; Wolff, 2002), motor tasks. An important aspect of our study is that because we controlled for ADHD symptomology, we can rule out ADHD as being the underlying reason for the between-group difference in the right anterior cerebellum. Note that Nicolson et al. (1999) did not study the left hand and we did not find between-group differences in activation during left hand tapping, suggesting the aberration in dyslexia is constrained to the right hand. While we did find differences in functional connectivity in dyslexia for both hands, these were in the cortex and did not involve the cerebellum. This observation is notable as it does not fit with cerebellar deficit hypothesis of dyslexia which emphasizes the connections from the cerebellum to the cortex, as discussed below. It is worth noting that our task was different from that used by Nicolson et al. (1999) where participants pressed one of four fingers in response to a pacing tone once every 3 s and feedback (a tone) was provided on whether the last press was accurate in terms of the sequence. Our task also involved externally-triggered motor movement, which is the most common approach to activation of the motor system for finger movements (Witt et al., 2008), but using a visual stimulus. Importantly, irregular pacing, as used here, elicits greater activation than regular pacing in the cerebellum, SM1, and SMA (Lutz et al., 2000). This task has previously been shown to elicit activation of the motor system in many studies, including one from our own lab (Turesky et al., 2018), and it elicited robust activation in the current study (including the cerebellum) for both groups and during the use of either hand. It is therefore unlikely that we did not find further differences (e.g., in the cortex) between our two groups because of the task.

In general, one might have expected any differences in dyslexia to be more widespread throughout the motor system, especially if they are causal to the reading problems. It is unlikely that we did not see more pervasive differences based on our participants, as our group with dyslexia was very impaired in their reading ability, scoring lower than the control group by more than three standard deviations on the measure of reading fluency and more than two and a half standard deviations on the measure of word identification. Further, as studies in children are more prone to artifacts related to head movement, we applied a strict protocol for data quality control leading to the exclusion of a large portion of our participants for the benefit of higher quality data. Future studies should therefore also aim for larger sample sizes than those used in the current study and in prior studies of the motor system in dyslexia.

Finally, when we tested for correlations between the signal in the right anterior cerebellum cluster with measures of reading, there were no significant results, making it difficult to attribute the reading difficulties that are the cardinal feature of dyslexia to this singular difference in activity in the motor system. It should be noted that the notion of dysfunction of the motor system, especially the cerebellum, in dyslexia has not enjoyed much support and has been criticized on theoretical grounds, as well as for concerns around “cerebellar treatment” for this reading disability (Zeffiro and Eden, 2001; McPhillips, 2003; Richards et al., 2003; Singleton and Stuart, 2003; Snowling and Hulme, 2003; Stein, 2003; Rack et al., 2007). Our results, too, do not offer any further evidence that would motivate treatment targeting the motor system for dyslexia. As noted by others in this debate, such an approach would not likely lead to a positive outcome, and could detract from the language-based literacy instruction that has shown efficacy.

### 4.3. Motor system functional connectivity in dyslexia

As part of our study, we examined functional connections between target seed regions placed in those areas activated during the finger tapping task (cerebellum, SM1, and SMA, specific for each hand) and the rest of the brain. We identified four functional connections involving left and right SM1 seeds that differed between children with and without dyslexia. These observations fit with prior work, which has shown anatomical anomalies in left (albeit not right) precentral gyrus (Krafnick et al., 2014). The left precentral gyrus has also been shown to be overactivated in those with dyslexia during reading, although in a more lateral, anterior, and inferior location on the gyrus (Richlan et al., 2011). Further, we observed greater iFC in the group with dyslexia between the left SM1 seed and left thalamus, which constitutes a segment of the cortico-cerebellar and cortico-striatal loops. Specifically, motor cortex projects to the anterior cerebellum as part of the cortico-cerebellar loop, passing through the pontine nucleus in the brainstem along its efferent segment and through the thalamus as part of its afferent segment (Kelly and Strick, 2003; for a review, see Ramnani, 2006). Our findings may suggest dysregulation of this loop, limited to left SM1 and left thalamus connection, a hypothesis bolstered by observed activity differences in the anterior cerebellum. Dysregulation in this segment is further evinced by an empirical diffusion tensor imaging (DTI) study in adolescents, which showed that children with dyslexia exhibited greater anatomical connectivity in thalamocortical tracts between thalamus and SM1 bilaterally compared with age-matched typical readers (Fan et al., 2014). In addition, Vandermosten et al. (2012) examined regions where white matter structure (i.e., functional anisotropy) correlated with reading ability and found (using probabilistic fiber tracking) that a large portion was within the left corona radiata (Vandermosten et al., 2012), which in general contains afferent fibers from thalamus to M1. Whether the iFC differences we observed are related to this white matter pathway specifically would need to be tested in future studies. Notably, we did not observe differences in cerebellar functional connectivity, which would have been expected based on proposed mechanisms by which the cerebellum affects language, namely via a disruption in cerebellar-frontal cortex connections (Nicolson and Fawcett, 2007).

It is also important to consider whether our observations can be explained specifically in the context of the motor system, or reflect general brain dysfunction underlying language that are reflected in structures that serve the motor systems, making the cerebellum an innocent bystander (Zeffiro and Eden, 2001). We addressed this issue by testing for brain-behavioral relationships between activation and functional connectivity estimates and measures of word reading accuracy and reading fluency, but we found no correlations. Broadly, this is consistent with previous behavioral literature showing no relationships between measurements of motor performance and reading deficits (Rochelle and Talcott, 2006) or ability (White et al., 2006). Many of the participants with and without dyslexia were also included in a study of brain activity and functional connectivity in the cerebellum during reading (Ashburn et al., 2020). This study found no between-group differences in activity during reading in the cerebellum, again suggesting that the cerebellum is not altered in ways that affect reading. It did, however, find more intrinsic functional connectivity between a seed region in right crus I and three left-hemisphere perisylvian target seed regions, angular gyrus, posterior superior temporal gyrus, and inferior frontal gyrus (Ashburn et al., 2020). A more recent resting-state iFC study by Greeley et al. (2021) found both weaker and stronger functional connectivity in a group of children with dyslexia (compared to a control group) between cerebellar right crus I, right lobule VI, and right lobule VIII seeds and widespread motor and non-motor regions of the cerebral cortex. Further, functional connections between right cerebellar lobule VIII and right frontal pole, and between cerebellar lobule VIII and left angular gyrus were positively related to reading measures in the group with dyslexia. However, these associations did not survive correction for multiple correction and there were no significant correlations with other regions of the cerebellum, including those associated with language (i.e., lobule VI, crus I, and crus II; Greeley et al., 2021). Other studies in children with dyslexia have examined iFC without including the cerebellum or without a finding in the cerebellum (Koyama et al., 2013; Horowitz-Kraus et al., 2018; Twait et al., 2018; Freedman et al., 2020). Overall, the lack of differences in brain-behavior relationships in the current study dovetail with previous studies, which have argued that the motor differences (whether brain or behavioral) may be epiphenomenal, but are not a defining feature of dyslexia (Ramus, 2003).

### 4.4. Conclusion

While dyslexia is associated with difficulties in reading due to language-based deficits in phonological processing, there has been a small and controversial body of literature focusing on deficits in motor function. Here, we found that children with dyslexia exhibited weaker activation in right anterior cerebellum during right hand tapping compared with the control group, without further differences. Our results also indicate compromise in some connections within the motor system, most notably between left SM1 and left thalamus during right hand finger tapping, but none with the cerebellum. As none of these findings showed a relationship with measures of reading ability, our results do not support the hypothesis that the motor system, specifically the cerebellum, has a critical role in reading.

## Data availability statement

The raw data supporting the conclusions of this article will be made available by the authors, without undue reservation.

## Ethics statement

The studies involving human participants were reviewed and approved by Georgetown University Institutional Review Board. All children were asked for their assent. Written informed consent to participate in this study was provided by the participants’ legal guardian/next of kin.

## Author contributions

GE conceived and designed the study. GE and ML were involved in overall study logistics and data collection. TT performed statistical analyses and together with GE drafted the manuscript. All authors contributed to the article and approved the submitted version.

## Funding

This work was supported by the Eunice Kennedy Shriver National Institute of Child Health and Human Development (P50 HD40095 and R01 HD056107), the National Institute of Neurological Disorders and Stroke’s Training in Neural Injury and Plasticity (T32 NS041218) and funds from the Intellectual and Developmental Disabilities Research Center (P30 HD040677) awarded to the Center for Functional and Molecular Imaging.

## Conflict of interest

The authors declare that the research was conducted in the absence of any commercial or financial relationships that could be construed as a potential conflict of interest.

## Publisher’s note

All claims expressed in this article are solely those of the authors and do not necessarily represent those of their affiliated organizations, or those of the publisher, the editors and the reviewers. Any product that may be evaluated in this article, or claim that may be made by its manufacturer, is not guaranteed or endorsed by the publisher.

## Footnotes

^1^https://www.fil.ion.ucl.ac.uk/spm/software/spm12/

^2^https://www.nitrc.org/artificat_detect

^3^http://marsbar.sourceforge.net/index.html

^4^https://www.nitrc.org/projects/conn

^5^https://www.nitrc.org/rex

^6^http://talairach.org/client.html

^7^http://www.talairach.org/applet.html

^8^http://lrnlab.org/

^9^http://rii.uthscsa.edu/mango/

## Supplementary material

The Supplementary material for this article can befound online at: https://www.frontiersin.org/articles/10.3389/fnins.2023.1135437/full#supplementary-material
