# Supplementary material for: An fMRI study of finger movements in children with and without dyslexia
Source: Front Neurosci. 2023 May 18;17:1135437. doi: 10.3389/fnins.2023.1135437 (PMC10233035; doi:10.3389/fnins.2023.1135437)
Supplement: Supplementary file 1 [file Table_1.docx]

| **Supplemental Table 1.** iFC peaks for within-group contrasts for cerebellum, SM1, and SMA seeds. | | | | | | | | |
| --- | --- | --- | --- | --- | --- | --- | --- | --- |
| **Group** | **Anatomical region** | **Functional motor region** | **BA** | **Peak MNI coordinate** | | | **k** | **Z** |
| *Seed* |  |  |  | **x** | **y** | **z** |  |  |
| **Control** |  |  |  |  |  |  |  |  |
| *Left Hand Tapping* | |  |  |  |  |  |  |  |
| *L. CB seed* | R. Postcentral Gyrus | R. SM1 | 2 | 42 | -30 | 66 | 170 | 3.82 |
|  | L. Medial Frontal Gyrus^#^ | R. SMA | 6 | 2 | -14 | 56 | 96 | 3.75 |
|  | L. Ant. Cerebellum |  |  | -18 | -58 | -16 | 2142 | 6.88 |
|  | L. Ant. Cerebellum |  |  | -54 | -56 | -32 | 113 | 4.49 |
|  |  |  |  |  |  |  |  |  |
| *R. SM1 seed* | R. Paracentral Lobule | R. SMA | 5 | 4 | -24 | 58 | 148 | 4.17 |
|  | L. Ant. Cerebellum |  |  | -22 | -48 | -22 | 77 | 3.78 |
|  | R. Precentral Gyrus | R. SM1 | 4 | 38 | -22 | 64 | 2091 | 6.74 |
|  |  |  |  |  |  |  |  |  |
| *R. SMA seed* | L. Postcentral Gyrus | L. SM1 | 3 | -46 | -12 | 60 | 1040 | 5.35 |
|  | L. Insula | L. PMv | 13 | -40 | 4 | 12 | 1268 | 5.18 |
|  | L. Precentral Gyrus | L. PMv | 4 | -62 | 2 | 16 | 120 | 4.69 |
|  | L. Lentiform Nucleus |  |  | -20 | -4 | -6 | 68 | 4.25 |
|  | R. Middle Temporal Gyrus |  | 39 | 56 | -68 | 32 | 162 | -4.09 |
|  | L. Middle Temporal Gyrus |  | 39 | -50 | -66 | 28 | 162 | -3.91 |
|  | L. Inferior Frontal Gyrus |  | 9 | -58 | 22 | 26 | 60 | -3.80 |
|  | L. Cingulate Gyrus |  | 31 | -4 | -42 | 38 | 242 | -5.10 |
|  | L. Medial Frontal Gyrus |  | 10 | 0 | 66 | 2 | 1465 | -4.82 |
|  | R. Precentral Gyrus |  | 6 | 68 | 8 | 18 | 1209 | 4.77 |
|  | L. Middle Frontal Gyrus |  | 6 | -32 | 12 | 58 | 93 | -4.54 |
|  | L. Post. Cingulate Gyrus |  | 31 | -12 | -64 | 20 | 111 | -3.86 |
|  | L. Post. Cerebellum |  |  | -24 | -74 | -16 | 54 | 4.27 |
|  | L. Medial Frontal Gyrus | R. SMA | 6 | 0 | -4 | 62 | 4255 | 6.95 |
|  |  |  |  |  |  |  |  |  |
| *Right Hand Tapping* | |  |  |  |  |  |  |  |
| *R. CB seed* | L. Inferior Parietal Lobule^⌿^ | L. SM1 | 40 | -40 | -26 | 44 | 619 | 4.39 |
|  | L. Sub-Gyral Temporal Lobe |  | 21 | -38 | -6 | -16 | 176 | 4.01 |
|  | R. Ant. Cerebellum |  |  | 10 | -54 | -14 | 2639 | 6.48 |
|  |  |  |  |  |  |  |  |  |
| *L. SM1 seed* | L. Medial Frontal Gyrus | L. SMA | 6 | -8 | -4 | 58 | 506 | 5.07 |
|  | L. Middle Temporal Gyrus |  | 21 | -60 | -28 | -6 | 82 | 4.72 |
|  | L. Medial Frontal Gyrus^#^ |  | 9 | 2 | 56 | 22 | 206 | 4.36 |
|  | L. Precentral Gyrus | L. SM1 | 4 | -38 | -20 | 60 | 2297 | 6.72 |
|  |  |  |  |  |  |  |  |  |
| *L. SMA seed* | R. Postcentral Gyrus | R. SM1 | 3 | 26 | -20 | 66 | 65 | 3.89 |
|  | R. Precentral Gyrus | R. PMd | 4 | 52 | -6 | 44 | 340 | 4.84 |
|  | L. Precentral Gyrus |  | 44 | -58 | 16 | 0 | 246 | 4.42 |
|  | L. Medial Frontal Gyrus | L. SMA | 6 | -2 | -2 | 62 | 3667 | 7.79 |
|  |  |  |  |  |  |  |  |  |
|  |  |  |  |  |  |  |  |  |
| **Dyslexia** |  |  |  |  |  |  |  |  |
| *Left Hand Tapping* | |  |  |  |  |  |  |  |
| *L. CB seed* | L. Postcentral Gyrus | L. SM1 | 2 | -66 | -18 | 30 | 98 | 4.19 |
|  | R. Postcentral Gyrus | R. SM1 | 2 | 28 | -32 | 76 | 49 | 4.17 |
|  | L. Middle Temporal Gyrus |  | 21 | -52 | -4 | -18 | 49 | 4.11 |
|  | L. Post. Cerebellum |  |  | -26 | -54 | -48 | 135 | 4.26 |
|  | L. Ant. Cerebellum |  |  | -18 | -54 | -18 | 1849 | 7.25 |
|  |  |  |  |  |  |  |  |  |
| *R. SM1 seed* | L. Postcentral Gyrus | L. SM1 | 3 | -34 | -18 | 52 | 88 | 4.30 |
|  | L. Postcentral Gyrus | L. SM1 | 2 | -38 | -32 | 68 | 58 | 4.05 |
|  | L. Ant. Cerebellum |  |  | -6 | -52 | -12 | 47 | 3.89 |
|  | L. Fusiform Gyrus |  | 37 | -60 | -60 | -18 | 394 | 4.70 |
|  | L. Subcallosal Gyrus |  | 34 | -12 | 4 | -20 | 55 | 4.72 |
|  | R. Post. Cerebellum* |  |  | 32 | -44 | -56 | 125 | 5.42 |
|  | L. Post. Cerebellum |  |  | -20 | -58 | -50 | 153 | 4.36 |
|  | L. Post. Cerebellum* |  |  | -26 | -44 | -54 | 134 | 4.11 |
|  | L. Post. Cerebellum |  |  | -34 | -88 | -18 | 138 | 4.00 |
|  | L. Post. Cerebellum |  |  | -10 | -74 | -42 | 100 | 3.84 |
|  | R. Lingual Gyrus |  | 18 | 14 | -84 | 6 | 352 | 4.64 |
|  | R. Precentral Gyrus | R. SM1 | 4 | 38 | -20 | 60 | 3006 | 6.39 |
|  |  |  |  |  |  |  |  |  |
| *R. SMA seed* | R. Postcentral Gyrus | R. SM1 | 2 | 52 | -22 | 60 | 176 | 4.07 |
|  | R. Postcentral Gyrus | R. SM1 | 3 | 36 | -30 | 58 | 90 | 3.88 |
|  | L. Superior Temporal Gyrus |  | 13 | -58 | -38 | 26 | 121 | 4.31 |
|  | R. Middle Frontal Gyrus |  | 10 | 46 | 48 | 2 | 126 | 4.70 |
|  | R. Precentral Gyrus |  | 44 | 54 | 4 | 0 | 124 | 4.33 |
|  | L. Post. Cerebellum |  |  | -34 | -78 | -50 | 116 | 5.13 |
|  | L. Medial Frontal Gyrus^#^ | R. SMA | 6 | 2 | -2 | 58 | 1404 | 6.70 |
|  |  |  |  |  |  |  |  |  |
| *Right Hand Tapping* | |  |  |  |  |  |  |  |
| *R. CB seed* | L. Postcentral Gyrus |  | 43 | -58 | -16 | 16 | 126 | 4.60 |
|  | L. Superior Temporal Gyrus |  | 38 | -36 | 0 | -16 | 314 | 4.40 |
|  | Midline Pons* |  |  | 0 | -16 | -28 | 57 | 4.09 |
|  | R. Post. Cerebellum |  |  | 12 | -60 | -14 | 2425 | 6.67 |
|  |  |  |  |  |  |  |  |  |
| *L. SM1 seed* | L. Precentral Gyrus | L. SM1 | 4 | -38 | -22 | 60 | 1102 | 7.03 |
|  |  |  |  |  |  |  |  |  |
| *L. SMA seed* | R. Postcentral Gyrus | R. SM1 | 3 | 42 | -26 | 56 | 553 | 4.47 |
|  | L. Inferior Parietal Lobule | L. SM1 | 40 | -52 | -24 | 52 | 60 | 3.78 |
|  | L. Insula | L. PMv | 13 | -40 | 4 | 14 | 68 | 4.28 |
|  | R. Superior Temporal Gyrus |  | 22 | 58 | 8 | -4 | 159 | 4.45 |
|  | R. Inferior Parietal Lobule |  | 40 | 60 | -22 | 26 | 56 | 4.24 |
|  | L. Medial Frontal Gyrus | L. SMA | 6 | -2 | -4 | 60 | 2546 | 6.85 |
| *Peak coordinate reported not within gray matter (11mm cube) by Talairach Client. Anatomical Label determined by visual inspection of nearest gray matter according to the MNI brain template in Mango. | | | | | | | | |
| ^⌿^Inconistence between anatomical and functional regions | | |  |  |  |  |  |  |
| ^#^Inconsistence between +/- x-coordinate and Talairach Client R/L identification | | | | | |  |  |  |
